# Cardiac fibrosis in mice expressing an inducible myocardial-specific Cre driver

**DOI:** 10.1242/dmm.010470

**Published:** 2013-08-07

**Authors:** Jonas Lexow, Tommaso Poggioli, Padmini Sarathchandra, Maria Paola Santini, Nadia Rosenthal

**Affiliations:** 1 National Heart and Lung Institute, Heart Science Centre and Hammersmith Hospital Campus, Imperial College London, London, W12 0NN, UK; 2 European Molecular Biology Laboratory (EMBL) Mouse Biology Unit, Via Ramarini 32, 00015 Monterotondo, Italy; 3 Australian Regenerative Medicine Institute/EMBL Australia, Monash University, Melbourne, 3800 VIC, Australia

## Abstract

Tamoxifen-inducible Cre-mediated manipulation of animal genomes has achieved wide acceptance over the last decade, with numerous important studies heavily relying on this technique. Recently, a number of groups have reported transient complications of using this protocol in the heart. In the present study we observed a previously unreported focal fibrosis and depressed left-ventricular function in tamoxifen-treated *αMHC-MerCreMer*-positive animals in a *Tβ4shRNAflox* × *αMHC-MerCreMer* cross at 6–7 weeks following standard tamoxifen treatment, regardless of the presence of the floxed transgene. The phenotype was reproduced by treating mice from the original *αMHC-MerCreMer* strain with tamoxifen. In the acute phase after tamoxifen treatment, cell infiltration into the myocardium was accompanied by increased expression of pro-inflammatory cytokines (*IL-1β*, *IL-6*, *TNFα*, *IFNγ*, *Ccl2*) and markers of hypertrophy (*ANF*, *BNP*, *Col3a1*). These observations highlight the requirement for including tamoxifen-treated *MerCreMer* littermate controls to avert misinterpretation of conditional mutant phenotypes. A survey of the field as well as the protocols presented here suggests that controlling the parameters of tamoxifen delivery is important in avoiding the chronic MerCreMer-mediated cardiac phenotype reported here.

## INTRODUCTION

A currently popular mode of conditionally manipulated gene expression in the mouse involves Cre-recombinase-mediated excision of engineered genomic target sequences flanked by *loxP* sites (reviewed in Smedley et al., 2011). Controlling Cre expression with tissue- or cell-specific promoters and drug-inducible cassettes allows spatial and temporal restriction of these recombination events. In the heart, inducible Cre-mediated excision driven by the cardiac-specific α-myosin heavy chain (*αMHC*) promoter has been extensively used to drive cardiomyocyte-restricted recombination of floxed genes in animal models. In *αMHC-MerCreMer* mice, Cre recombinase is fused to two mutated estrogen receptor domains (Mer); binding to the estrogen analog tamoxifen shuttles the fusion protein from the cytoplasm to the nucleus (Zhang et al., 1996; Sohal et al., 2001; Nakamura et al., 2006). *αMHC-MerCreMer* mice have found wide application in conditional knockout/knockdown and lineage tracing experiments (Hsieh et al., 2007). However, recent reports have indicated that tamoxifen-priming of mice carrying the cardiomyocyte-specific MerCreMer cassette causes dose-dependent acute cardiac dysfunction such as decreased left-ventricular fractional shortening within the first week following tamoxifen exposure and mitochondrial aberrations until 14 days post-treatment (Hall et al., 2011). Transient effects were shown to include substantial changes in the transcriptome (Hougen et al., 2010), further driving current efforts to achieve maximal recombination with a minimum of cardiac side effects, mainly by reducing the total dose of tamoxifen that is delivered (Andersson et al., 2010). Here, we undertake a systematic morphological and molecular evaluation of Cre-recombinase induction in the mouse heart, using standard reagents and protocols, and present a protocol that avoids these pathological responses. Our detailed characterization of pathological effects of Cre induction on cardiac morphology, physiology and function affords a comparative analysis of published results and highlights the importance of transgenic background when interpreting conditional mutant cardiac phenotypes.

## RESULTS

**Fig. 1.**
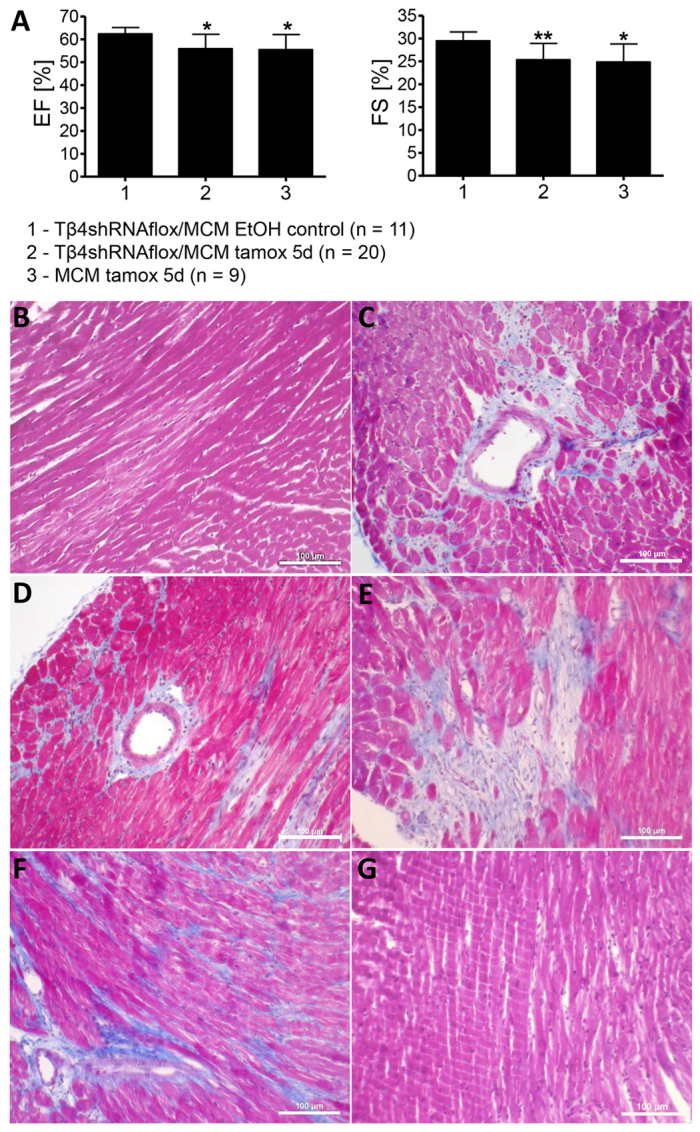
**Echocardiography analysis of mouse hearts 6–7 weeks after completing 5 days of tamoxifen injections (20 mg/kg body weight IP daily)**. (A) Ejection fraction (EF) measurements were recorded in 2D-mode and fractional shortening (FS) in M-mode. Control-injected (EtOH) *Tβ4shRNAflox/MerCreMer* (MCM) double transgenic mice served as controls. *P<0.05, **P<0.01. (B) Normal cardiac tissue in tamoxifen-treated wild-type and *Tβ4shRNAflox* animals and control-treated *Tβ4shRNAflox/MerCreMer* mice. (C,D) Representative images of the extensive cardiac fibrosis and cardiomyocyte replacement observed in ∼60% of tamoxifen-treated *Tβ4shRNAflox/MerCreMer* (C) and *αMHC-MerCreMer* (D) littermates. (E,F) Interstitial (E) and perivascular (F) fibrosis in the original *αMHC-MerCreMer* strain after 5 days tamoxifen treatment and 1 month of rest. (G) Normal cardiac morphology observed in *αMHC-MerCreMer* mice following a single injection of tamoxifen (40 mg/kg body weight).

In previous studies of inducible Cre-mediated mouse mutagenesis, tamoxifen administered by intra-peritoneal (IP) injection ranged from 40 mg/kg to 560 mg/kg body weight (Nakai et al., 2007; Wang et al., 2009; Stokke et al., 2010) delivered over periods of 1–14 days. In the current study, we induced MerCreMer-mediated excision of an upstream inhibitory sequence by using a standard injection protocol of one injection per day of 20 mg tamoxifen/kg body weight (100 mg/kg total) for 5 days to drive expression of an shRNA specifically in cardiomyocytes (Sohal et al., 2001) to knock down cardiac thymosin β4 (*Tβ4*) in adult mice. Echocardiography measurements at 6–7 weeks after tamoxifen injection of mice harboring an *αMHC-MerCreMer* transgene, with or without the *Tβ4shRNAflox* target allele, indicated decreased cardiac function in a significant portion of injected mice [*Tβ4shRNAflox/MerCreMer* % ejection fraction (EF) 55.90±6.29, % fractional shortening (FS) 25.35±3.57; *αMHC-MerCreMer* %EF 55.52±6.58, %FS 24.86±3.93, compared with control-injected *Tβ4shRNAflox/MerCreMer* %EF 62.36±2.76, %FS 29.49±1.93; Fig. 1A]. Histological analysis revealed that ∼60% (*n*=17/28) of tamoxifen-treated *αMHC-MerCreMer*-positive animals presented significant cardiac fibrosis regardless of the presence of the floxed target gene (Fig. 1C,D). Fibrosis was patchy and present in the left ventricle, septum and right ventricle, and was not observed in *Tβ4shRNAflox* or wild-type (wt) littermates (*n*=5) or in control-treated *Tβ4shRNAflox/MerCreMer* mice (*n*=10), all of which showed normal cardiac morphology (Fig. 1B).

**TRANSLATIONAL IMPACT**

**Clinical issue**

Cardiac fibrosis, which is characterized by excessive proliferation of cardiac fibroblasts and reduced contractility of the heart, is a hallmark of cardiomyocyte death during congenital or acquired heart disease. The detrimental effects of fibrosis on organ architecture and function can eventually lead to cardiac failure. Mouse models of human heart disease frequently involve the use of a genetic ‘switch’ that induces Cre recombinase activity in specific cardiac cells, thereby modifying expression of the gene under study in the living heart. However, the Cre driver itself might be a source of cardiac fibrosis, underscoring the need to refine the genetic manipulation and more clearly define any associated cardiac phenotype.

**Results**

Here, the authors systematically characterized the effects of Cre recombinase induction on cardiac morphology, physiology and function in mice. Tamoxifen was used to induce MerCreMer-mediated excision of cardiomyocyte-specific floxed cassettes, in line with standard protocols. Histological analysis revealed that ∼60–100% of tamoxifen-treated Cre-driver mice displayed cardiac fibrosis (with variation in penetrance in different genetic backgrounds) independent of the presence of the floxed target gene. Moreover, echocardiography revealed significant depression of cardiac function in this group. The authors also reported elevated expression of pro-inflammatory cytokines following tamoxifen treatment of Cre-driver mice. In contrast to standard multiple-injection protocols, cardiac function was not perturbed in Cre-driver mice treated with a single tamoxifen injection to induce efficient recombination of the target gene.

**Implications and future directions**

The present study provides evidence for long-term pathological damage and compromised heart function caused by MerCreMer recombinase induction in heart tissue. These findings are consistent with prior reports of the negative impacts of tamoxifen exposure on cardiac function; however, previous studies suggested that the effects are transient. In addition, the group showed that differences in the genetic background can contribute to the observed variation in phenotypic penetrance. Thus, the adverse long-term side effects of Cre recombinase induction in the heart must be carefully monitored in mouse models of cardiac disease involving gene switching. The authors show that a single injection protocol can overcome the toxic effects associated with the method, and recommend the use of this or other delivery methods to minimize MerCreMer-mediated cardiac damage and thereby optimize the use of these mice as models for cardiac disease.

**Fig. 2.**
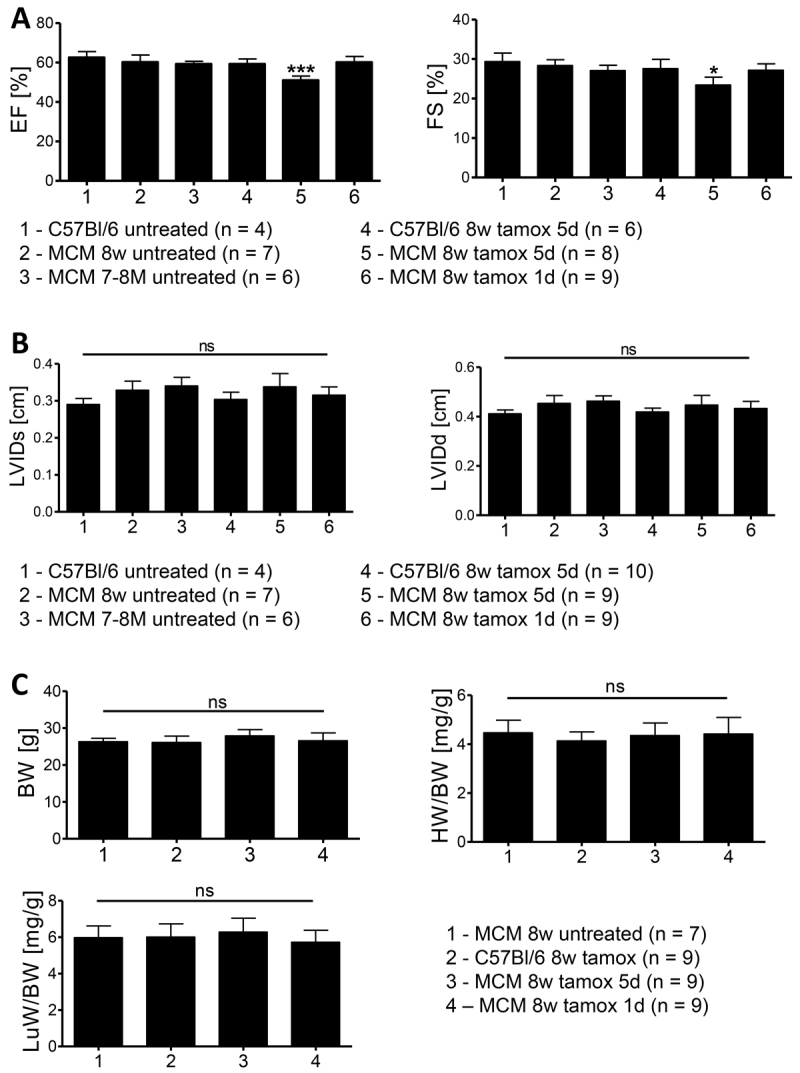
**Assessment of treating 8-week-old *αMHC-MerCreMer* mice with tamoxifen.** (A) Echocardiography analysis of mouse hearts 1 month after completing 5 days of tamoxifen injections (20 mg/kg body weight IP daily) compared with untreated wild-type and *αMHC-MerCreMer* (MCM) animals of various ages, tamoxifen-treated *αMHC-MerCreMer*-negative (*C57BL/6*) littermates and *αMHC-MerCreMer* animals treated with a single injection of 40 mg tamoxifen/kg body weight. Ejection fraction (EF) and fractional shortening (FS) measurements were recorded in 2D-mode and M-mode, respectively. *P<0.05, *P*<0.001. (B) Left-ventricular inner diameters during systole (LVIDs) and diastole (LVIDd) calculated from M-mode recordings. (C) Body weight (BW), relative heart weight (HW/BW) and relative lung weight (LuW/BW) recorded 1 month following tamoxifen or control treatment. Not significant (ns): *P*>0.05.

Because high levels of Cre are reportedly cytotoxic, we analyzed *Cre* copy numbers in fibrotic and normal mice (Buerger et al., 2006). Significant fibrosis was observed in both homozygous and heterozygous *αMHC-MerCreMer* mice (12/19 heterozygotes and 5/8 homozygotes affected). To address the possibility of strain-specific effects of *MerCreMer* expression in our crosses (*129Ola/C57Bl6/J* mixed background), we examined 8-week-old heterozygous *αMHC-MerCreMer* on *C57Bl/6J* background 1 month following tamoxifen treatment. We observed a significant depression of cardiac function by echocardiography (%EF 51.04±2.10, %FS 23.40±2.00), compared with wt mice (%EF 62.55±3.00, %FS 29.35±2.17), untreated *αMHC-MerCreMer* mice of various ages (8 weeks %EF 60.21±3.64, %FS 28.36±1.44, 7–8 months %EF 59.27±1.34, %FS 27.07±1.35) and tamoxifen-treated *MerCreMer*^−/−^ littermates (%EF 59.31±2.50, %FS 27.55±2.37; Fig. 2A), as well as interstitial and perivascular cardiac fibrosis (5/5; Fig. 1E,F). Cardiac hypertrophy was not observed, with normal inner ventricular dimensions measured across all groups (Fig. 2B). Furthermore, relative heart or lung weights, secondary indicators of hypertrophy or heart failure, were unchanged (Fig. 2C).

**Fig. 3.**
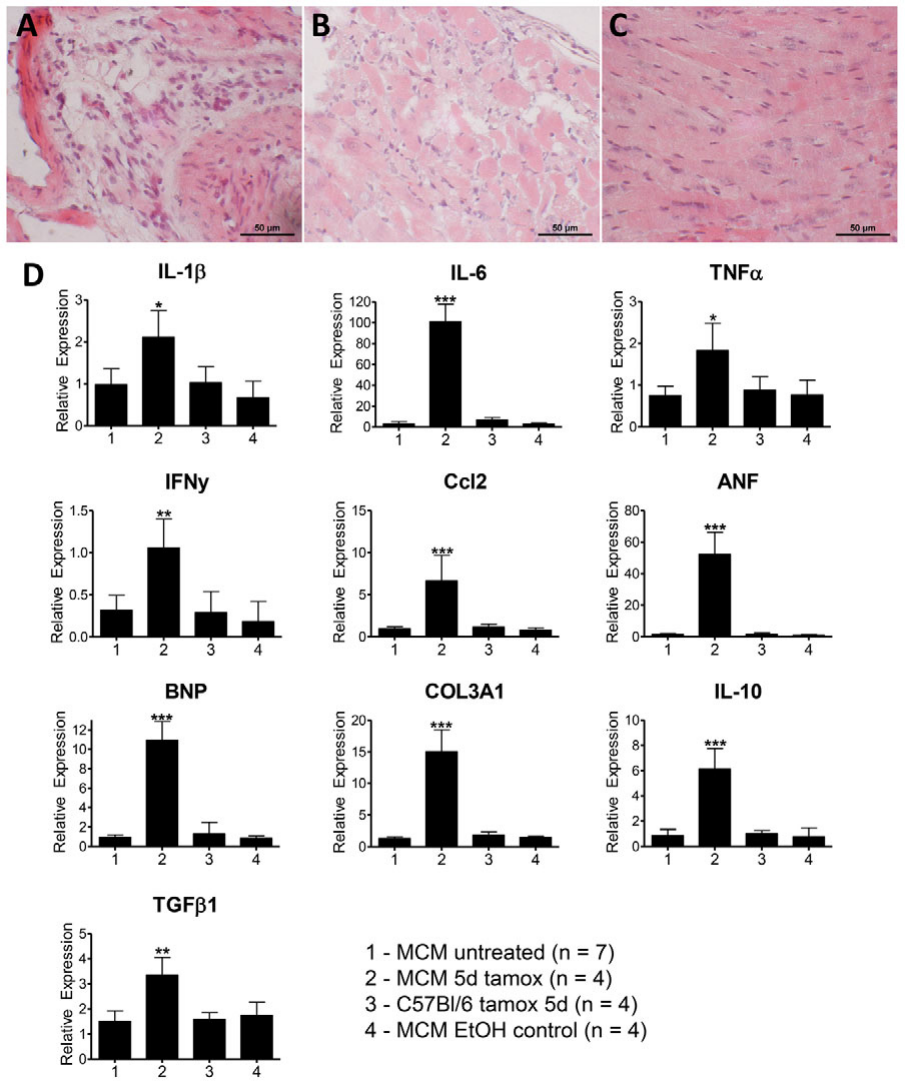
**Inflammatory response in *αMHC-MerCreMer* mice 3 days after 5 daily tamoxifen injections (20 mg/kg body weight).** (A–C) H&E staining reveals cell infiltrates in severely affected areas (A,B) and normal morphology in healthy regions (C) in cardiac sections of tamoxifen-treated *αMHC-MerCreMer* mice. (D) Real-time PCR analysis of whole heart RNA extracts following 5 days of tamoxifen (20 mg/kg body weight IP daily) or control injections and 3 days of rest. *P<0.05, **P<0.01, *P*<0.001.

To further investigate the cause of the phenotype, we analyzed histological sections of *αMHC-MerCreMer* hearts 3 days after tamoxifen treatment and identified focal cell infiltrates in the myocardium (Fig. 3A,B). We also performed real-time PCR (rtPCR) analyses of cytokines 3 days following tamoxifen treatment to study the contribution of inflammation to the observed phenotype. Pro-inflammatory markers (*IL1β*, *IL6*, *TNFα*, *TFNγ* and *Ccl2*) were significantly elevated in tamoxifen-treated animals compared with controls, as were markers of cardiac remodeling (*ANF*, *BNP* and *Col3A1*) (Fig. 3D). Interestingly, expression of the anti-inflammatory cytokines *IL-10* and *TGFβ* was also induced at this time point.

**Fig. 4.**
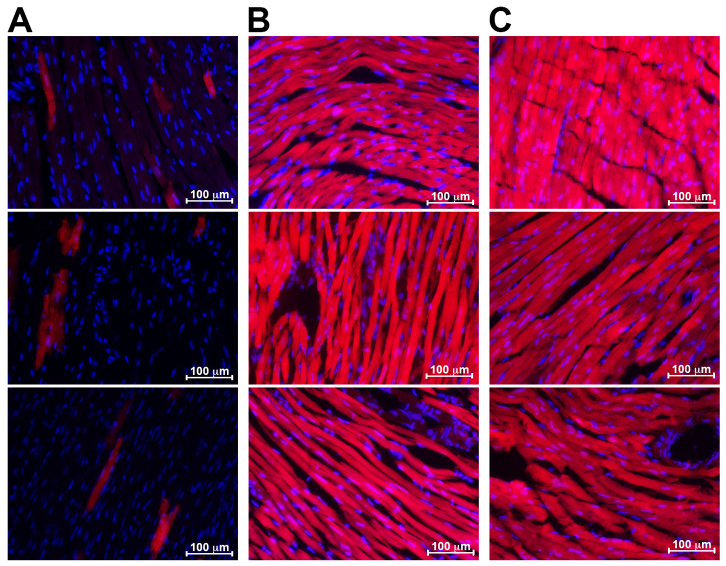
**A single tamoxifen injection is sufficient to induce robust *αMHC-MerCreMer* recombination.** 8-week-old *αMHC-MerCreMer* × *tdTomato mice* were injected with (A) control (EtOH), (B) a single dose of tamoxifen (40 mg/kg body weight) or (C) 5 daily tamoxifen injections (20 mg/kg body weight) and analyzed 2 weeks after injection. DAPI (blue) and tdTomato (red) staining shows background recombination in various regions of control heart tissue (A), and complete recombination in cardiomyocyte fibers but not in other cell types of induced mice regardless of injection protocol (B,C). A separate mouse was analyzed for each panel of B and C.

Because Hougen et al. proposed the use of a single injection of tamoxifen (40 mg/kg) to avoid a transient Cre-mediated cardiomyopathy, we investigated the suitability of this protocol to prevent the chronic defects observed following the 5-day protocol (Hougen et al., 2010). We observed normal cardiac function (%EF 60.14±2.92, %FS 27.16±1.62) and cardiac morphology on the microscopic scale in 8-week-old *αMHC-MerCreMer* mice 1 month after the single injection protocol (*n*=9) (Fig. 2A; Fig. 1G). To determine whether a single injection would induce efficient Cre-mediated recombination, *αMHC-MerCreMer* mice were crossed with a tdTomato/Ai14 reporter line (Madisen et al., 2010) and the hearts of 8-week-old animals were analyzed 2 weeks after either a single tamoxifen injection (40 mg/kg) or the 5-day consecutive injections (20 mg/kg). As seen in Fig. 4, whereas control mice injected with vehicle (EtOH) showed low levels of spontaneous Cre recombination that have been seen elsewhere (Kemp et al., 2004, Liu et al., 2010), all mice injected with tamoxifen showed uniform recombination in cardiomyocyte fibers, irrespective of the injection protocol. We conclude that a single injection of tamoxifen at 40 mg/kg effectively avoids the cardiomyopathological effects of prolonged drug exposure in *αMHC-MerCreMer* mice.

## DISCUSSION

In this report we show that episodic tamoxifen-primed nuclear Cre recombinase accumulation in adult hearts causes irreversible damage to the myocardium, reflected in decreased left-ventricular function. Because a single injection of tamoxifen (40 mg/kg) induced efficient Cre-mediated recombination but did not result in cardiac fibrosis in *αMHC-MerCreMer* mice, the observed phenotype presumably depends either on the nuclear levels of MerCreMer protein or its duration in the nucleus, or a combination of both. This possibility is supported by the observation of normal cardiac function and morphology in aged untreated animals, indicating that cytoplasmic MerCreMer itself is not toxic. Tamoxifen alone was also shown to have no effect in any *MerCreMer*-negative background.

The toxicity of nuclear Cre could potentially stem from promiscuous aspecific genomic recombination at cryptic *loxP* sites on the genome (Loonstra et al., 2001; Semprini et al., 2007), in which case chromosomal aberration from DNA fragmentation could be responsible for cell death and subsequent replacement of cardiac myocytes by fibroblasts, resulting in the observed phenotypes. The observed cell infiltration and upregulation of various cytokines involved in acute inflammation in the tamoxifen-treated *αMHC-MerCreMer* group reported here is a further indication of the negative impact of excessive active MerCreMer in cardiac tissue. Although this response could be secondary to excessive tissue damage or necrosis, it is a further factor potentially compromising experimental interpretation, as inflammatory response and cell death constitute primary readouts in cardiac regeneration research. This also applies for markers of cardiac hypertrophy (*ANF*, *BNP*, *Col3a1*), frequently used as indicators of hypertrophy in cardiac-specific conditional knockout mice. Elevated pro-hypertrophic markers in the *αMHC-MerCreMer* model have been reported previously, although higher doses of tamoxifen were used in these studies (40 mg/kg daily for 4 days, 160 mg total) (Andersson et al., 2010). The fact that *IL-10* and *TGFβ* mRNA levels are increased after 3 days is likely a negative feedback response and indicates the end of the acute inflammatory reaction following MerCreMer-mediated insult.

Our findings are consistent with recent studies reporting negative impacts of Cre and/or tamoxifen administration on animal physiology (Bersell et al., 2013). A survey of the literature revealed a number of recent studies investigating phenotypes of *αMHC-MerCreMer* cross-bred animals without including tamoxifen-treated *αMHC-MerCreMer* single-genotype controls. Among the reported phenotypes were decreased cardiac function, cardiac fibrosis, and increased expression of *ANF* and *BNP*. In light of our results these observations would have potentially been confounded by a Cre-mediated cardiomyopathy. Through personal communications with the authors of studies in which tamoxifen-treated *αMHC-MerCreMer* mice were not included, we found that, indeed, in most cases the control mice had been monitored, but were excluded from the published experiments owing to the absence of any remarkable phenotype. Differences in genetic background could be a contributing factor to the observed variation in phenotypic penetrance, which is supported by the fact that we observed fibrosis in 60% of *Tβ4shRNAflox/MerCreMer* mice (*129Ola/C57Bl6/J*) and in 100% of *αMHC-MerCreMer* mice (*C57Bl/6J*), following tamoxifen treatment.

The transient effects of tamoxifen exposure on cardiac function have been well documented, and methods have been proposed to reduce these effects (Hougen et al., 2010). In addition to the injection protocol, the form of tamoxifen used might constitute a parameter contributing to diverse outcomes. As reported previously by Kiermayer et al., tamoxifen citrate seems to be equally as effective and a potentially safer alternative to tamoxifen or its active metabolite 4-hydroxytamoxifen, but the oral delivery proposed is sensitive to diet refusal by the animals, resulting in insufficient recombination, weight loss and starvation-related phenotypes (Kiermayer et al., 2007; Andersson et al., 2009) (and our unpublished data). Consequently, injecting tamoxifen citrate could prove to be a suitable alternative (Bersell et al., 2013).

In summary, several conclusions can be drawn from the present report and a survey of the current literature. First, high levels of tamoxifen-induced MerCreMer can cause substantial cardiac phenotypes with short- and long-term effects. Second, genetic background is most likely a determining factor of the tamoxifen-induced MerCreMer phenotype. Third, delivery methods have varying effectiveness in terms of tamoxifen bioavailability, and consequently affect concentration and duration of MerCreMer in the nucleus. Therefore, the most appropriate delivery method for each model must be evaluated prior to extensive experimentation and the optimal dose of tamoxifen adjusted to the lowest concentration required for achieving sufficient recombination, as recently reported (Touvron et al., 2012).

Given the potential of tamoxifen treatment to cause a substantial phenotype in *MerCreMer* mice at molecular, macrostructural and functional levels, we strongly support the inclusion of tamoxifen-treated *MerCreMer* single-genotype animals as controls in relevant studies, particularly in experiments involving myocardial-specific *MerCreMer* transgenes (Anastassiadis et al., 2010). Because we show that the observed phenotype is not related to cytoplasmic MerCreMer or the presence of a floxed transgene, tamoxifen-treated *αMHC-MerCreMer* animals should be chosen over *flox/flox* mice as negative controls in studies in which time and resources prohibit the use of all applicable control groups.

## MATERIALS AND METHODS

### Animals

All animal work was performed in accordance with Imperial College London, UK institutional guidelines. *Tβ4shRNAflox* animals were kindly provided by Paul Riley from University College London, UK. *αMHC-MerCreMer* animals were purchased from The Jackson Laboratory (JAX stock number 005657). Male *Tβ4shRNAflox/MerCreMer* mice at 3–4 months of age and *αMHC-MerCreMer* mice between 8 weeks and 8 months were used in this study. The *tdTomato/Ai14* reporter line (Madisen et al., 2010) was a gift from Michael D. Schneider, Imperial College London, UK.

### *MerCreMer* induction

Tamoxifen (Sigma-Aldrich, Gillingham, UK, # T5648) was dissolved in ethanol (100%) to a concentration of 100 mg/ml and further diluted in soybean oil (Sigma-Aldrich, Gillingham, UK, # S7381) to 10 mg/ml (Nakamura et al., 2006). Tamoxifen solution was injected IP (20 mg/kg body weight) once daily for 5 consecutive days or as a single injection of 40 mg/kg body weight (Sohal et al., 2001; Hougen et al., 2010). EtOH in soybean oil at identical concentrations was injected as control. Animals were maintained for 6–7 weeks (*Tβ4shRNAflox/MerCreMer* study) or 1 month (*αMHC-MerCreMer* study) before sacrifice.

### Echocardiography analysis

Mice were maintained under low anesthesia [1.5% (v/v) isofluorane, 1.5 ml/minute O_2_]. Short-axis view transthoracic echocardiography (ECHO) was performed on shaved mice at midpapillary level. Ejection fraction (EF) was determined in 2D and M-mode; fractional shortening (FS) and l dimensions were measured in M-mode using a 12.5 MHz transducer (Siemens Acuson Sequoia C256). Each animal was analyzed at least in duplicate on consecutive days and results averaged. All raw ECHO data was recorded within 10 minutes from induction of anesthesia. At baseline, all groups showed indistinguishable normal cardiac function. ECHO data are shown as ± standard deviation (s.d.) of the mean.

### Immunohistochemistry

Heart samples were incubated in formal saline (10%) overnight at 4°C and embedded in paraffin the following day using standard protocols. Staining for collagen was performed using a Trichrome (Masson’s) kit (Sigma-Aldrich, Gillingham, UK, # HT15-1KT) following the manufacturer’s instructions. Hematoxylin and eosin (H&E) staining to investigate cell infiltration and morphology was performed using standard protocols. For the *tdTomato/Ai14* reporter experiment, hearts were fixed for 4 hours in PFA (4%) at room temperature, incubated overnight at 4°C in sucrose (10%) and embedded in OCT using standard protocols. Frozen sections were stained with 4′,6-diamidino-2-phenylindole (DAPI) for nuclei detection and examined for positive tdTomato staining induced by Cre recombination.

### Real-time PCR

Total RNA from heart was isolated 3 days following 5 days of tamoxifen or control injections. Comparative ΔC_T_ method was used to identify relative changes in mRNA expression using Taqman® reagents and an ABI7500 fast thermocycler (Applied Biosystems, Carlsbad, CA). 18s ribosomal subunit expression served as an endogenous expression control. The following transcripts were analyzed: *CCL2* (Mm00441242_m1), *COL3A1* (Mm01254476_m1), *Nppa* (Mm01255747_g1), *Nppb* (Mm01255770_g1), *IL-1β* (Mm00434189_m1), *IL-6* (Mm00446190_m1), *IL-10* (Mm00439614_m1), *IFNγ* (Mm01168134_m1), *TGFβ1* (Mm01178820_m1) and *TNFα* (Mm00443260_g).

### Copy number analysis

Genomic DNA was isolated from tails samples using a DNeasy kit (Qiagen, Crawley, UK). *Cre* copy abundance was determined by real-time PCR using a Taqman® probe targeting *Cre* cDNA (Mr00635245_cn).

### Statistical analysis

Experimental groups were compared by one-way analysis of variance (ANOVA) and Bonferroni post-testing. Results where *P*<0.05 were considered statistically significant (^*^). Error bars indicate s.d.

## References

[b1-0061470] Anastassiadis K. Glaser S. Kranz A. Berhardt K. Stewart A. F. (2010). A practical summary of site-specific recombination, conditional mutagenesis, and tamoxifen induction of CreERT2. Methods Enzymol. 477, 109–123 20699139 10.1016/S0076-6879(10)77007-5

[b2-0061470] Andersson K. B. Birkeland J. A. Finsen A. V. Louch W. E. Sjaastad I. Wang Y. Chen J. Molkentin J. D. Chien K. R. Sejersted O. M. (2009). Moderate heart dysfunction in mice with inducible cardiomyocyte-specific excision of the Serca2 gene. J. Mol. Cell. Cardiol. 47, 180–187 19328205 10.1016/j.yjmcc.2009.03.013

[b3-0061470] Andersson K. B. Winer L. H. Mørk H. K. Molkentin J. D. Jaisser F. (2010). Tamoxifen administration routes and dosage for inducible Cre-mediated gene disruption in mouse hearts. Transgenic Res. 19, 715–725 19894134 10.1007/s11248-009-9342-4

[b4-0061470] Bersell K. Choudhury S. Mollova M. Polizzotti B. D. Ganapathy B. Walsh S. Wadugu B. Arab S. Kühn B. (2013). Moderate and high amounts of tamoxifen in *αMHC-MerCreMer* mice induce a DNA damage response, leading to heart failure and death. Dis. Model. Mech. 6, 1459–1469 10.1242/dmm.010447 PMC3820268 23929941

[b5-0061470] Buerger A. Rozhitskaya O. Sherwood M. C. Dorfman A. L. Bisping E. Abel E. D. Pu W. T. Izumo S. Jay P. Y. (2006). Dilated cardiomyopathy resulting from high-level myocardial expression of Cre-recombinase. J. Card. Fail. 12, 392–398 16762803 10.1016/j.cardfail.2006.03.002

[b6-0061470] Hall M. E. Smith G. Hall J. E. Stec D. E. (2011). Systolic dysfunction in cardiac-specific ligand-inducible MerCreMer transgenic mice. Am. J. Physiol. 301, H253–H260 10.1152/ajpheart.00786.2010 PMC3129917 21536850

[b7-0061470] Hougen K. Aronsen J. M. Stokke M. K. Enger U. Nygard S. Andersson K. B. Christensen G. Sejersted O. M. Sjaastad I. (2010). Cre-loxP DNA recombination is possible with only minimal unspecific transcriptional changes and without cardiomyopathy in Tg(alphaMHC-MerCreMer) mice. Am. J. Physiol. 299, H1671–H1678 10.1152/ajpheart.01155.2009 20802136

[b8-0061470] Hsieh P. C. Segers V. F. Davis M. E. MacGillivray C. Gannon J. Molkentin J. D. Robbins J. Lee R. T. (2007). Evidence from a genetic fate-mapping study that stem cells refresh adult mammalian cardiomyocytes after injury. Nat. Med. 13, 970–974 17660827 10.1038/nm1618 PMC2754571

[b9-0061470] Kemp R. Ireland H. Clayton E. Houghton C. Howard L. Winton D. J. (2004). Elimination of background recombination: somatic induction of Cre by combined transcriptional regulation and hormone binding affinity. Nucleic Acids Res. 32, e92 15247325 10.1093/nar/gnh090 PMC443557

[b10-0061470] Kiermayer C. Conrad M. Schneider M. Schmidt J. Brielmeier M. (2007). Optimization of spatiotemporal gene inactivation in mouse heart by oral application of tamoxifen citrate. Genesis 45, 11–16 17216603 10.1002/dvg.20244

[b11-0061470] Liu Y. Suckale J. Masjkur J. Magro M. G. Steffen A. Anastassiadis K. Solimena M. (2010). Tamoxifen-independent recombination in the RIP-CreER mouse. PLoS ONE 5, e13533 21063464 10.1371/journal.pone.0013533 PMC2965077

[b12-0061470] Loonstra A. Vooijs M. Beverloo H. B. Allak B. A. van Drunen E. Kanaar R. Berns A. Jonkers J. (2001). Growth inhibition and DNA damage induced by Cre recombinase in mammalian cells. Proc. Natl. Acad. Sci. USA 98, 9209–9214 11481484 10.1073/pnas.161269798 PMC55399

[b13-0061470] Madisen L. Zwingman T. A. Sunkin S. M. Oh S. W. Zariwala H. A. Gu H. Ng L. L. Palmiter R. D. Hawrylycz M. J. Jones A. R. (2010). A robust and high-throughput Cre reporting and characterization system for the whole mouse brain. Nat. Neurosci. 13, 133–140 20023653 10.1038/nn.2467 PMC2840225

[b14-0061470] Nakai A. Yamaguchi O. Takeda T. Higuchi Y. Hikoso S. Taniike M. Omiya S. Mizote I. Matsumura Y. Asahi M. (2007). The role of autophagy in cardiomyocytes in the basal state and in response to hemodynamic stress. Nat. Med. 13, 619–624 17450150 10.1038/nm1574

[b15-0061470] Nakamura E. Nguyen M. T. Mackem S. (2006). Kinetics of tamoxifen-regulated Cre activity in mice using a cartilage-specific CreER(T) to assay temporal activity windows along the proximodistal limb skeleton. Dev. Dyn. 235, 2603–2612 16894608 10.1002/dvdy.20892

[b16-0061470] Semprini S. Troup T. J. Kotelevtseva N. King K. Davis J. R. Mullins L. J. Chapman K. E. Dunbar D. R. Mullins J. J. (2007). Cryptic loxP sites in mammalian genomes: genome-wide distribution and relevance for the efficiency of BAC/PAC recombineering techniques. Nucleic Acids Res. 35, 1402–1410 17284462 10.1093/nar/gkl1108 PMC1865043

[b17-0061470] Smedley D. Salimova E. Rosenthal N. (2011). Cre recombinase resources for conditional mouse mutagenesis. Methods 53, 411–416 21195764 10.1016/j.ymeth.2010.12.027

[b18-0061470] Sohal D. S. Nghiem M. Crackower M. A. Witt S. A. Kimball T. R. Tymitz K. M. Penninger J. M. Molkentin J. D. (2001). Temporally regulated and tissue-specific gene manipulations in the adult and embryonic heart using a tamoxifeninducible Cre protein. Circ. Res. 89, 20–25 11440973 10.1161/hh1301.092687

[b19-0061470] Stokke M. K. Hougen K. Sjaastad I. Louch W. E. Briston S. J. Enger U. H. Andersson K. B. Christensen G. Eisner D. A. Sejersted O. M. (2010). Reduced SERCA2 abundance decreases the propensity for Ca2+ wave development in ventricular myocytes. Cardiovasc. Res. 86, 63–71 20019150 10.1093/cvr/cvp401

[b20-0061470] Touvron M. Escoubet B. Mericskay M. Angelini A. Lamotte L. Santini M. P. Rosenthal N. Daegelen D. Tuil D. Decaux J. F. (2012). Locally expressed IGF1 propeptide improves mouse heart function in induced dilated cardiomyopathy by blocking myocardial fibrosis and SRF-dependent CTGF induction. Dis. Model. Mech. 5, 481–491 22563064 10.1242/dmm.009456 PMC3380711

[b21-0061470] Wang D. Patel V. V. Ricciotti E. Zhou R. Levin M. D. Gao E. Yu Z. Ferrari V. A. Lu M. M. Xu J. (2009). Cardiomyocyte cyclooxygenase-2 influences cardiac rhythm and function. Proc. Natl. Acad. Sci. USA 106, 7548–7552 19376970 10.1073/pnas.0805806106 PMC2670242

[b22-0061470] Zhang Y. Riesterer C. Ayrall A. M. Sablitzky F. Littlewood T. D. Reth M. (1996). Inducible site-directed recombination in mouse embryonic stem cells. Nucleic Acids Res. 24, 543–548 8604292 10.1093/nar/24.4.543 PMC145690

